# Estimation of the Burden of Serious Human Fungal Infections in Malaysia

**DOI:** 10.3390/jof4010038

**Published:** 2018-03-19

**Authors:** Rukumani Devi Velayuthan, Chandramathi Samudi, Harvinder Kaur Lakhbeer Singh, Kee Peng Ng, Esaki M. Shankar, David W. Denning

**Affiliations:** 1 Department of Medical Microbiology, Faculty of Medicine, University of Malaya, Kuala Lumpur 50603, Malaysia; chandramthi@ummc.edu.my (C.S.); harvinder@ummc.edu.my (H.K.L.S.); dr_kpng@hotmail.com (K.P.N.); 2 Centre of Excellence for Research in AIDS (CERiA), Faculty of Medicine, University of Malaya, Kuala Lumpur 50603, Malaysia; shankarem@cutn.ac.in; 3 Department of Microbiology, School of Basic & Applied Sciences, Central University of Tamil Nadu (CUTN), Thiruvarur 610 101, Tamil Nadu, India; 4 Faculty of Biology, Medicine and Health, The University of Manchester and Manchester Academic Health Science Centre, Oxford Road, Manchester M13 9PL, UK; ddenning@manchester.ac.uk; 5 The National Aspergillosis Centre, Education and Research Centre, Wythenshawe Hospital, Manchester M23 9LT, UK

**Keywords:** AIDS, aspergillosis, cryptococcal meningitis, epidemiology, HIV infection, Malaysia

## Abstract

Fungal infections (mycoses) are likely to occur more frequently as ever-increasingly sophisticated healthcare systems create greater risk factors. There is a paucity of systematic data on the incidence and prevalence of human fungal infections in Malaysia. We conducted a comprehensive study to estimate the burden of serious fungal infections in Malaysia. Our study showed that recurrent vaginal candidiasis (>4 episodes/year) was the most common of all cases with a diagnosis of candidiasis (*n* = 501,138). Oesophageal candidiasis (*n* = 5850) was most predominant among individuals with HIV infection. Candidemia incidence (*n* = 1533) was estimated in hospitalized individuals, some receiving treatment for cancer (*n* = 1073), and was detected also in individuals admitted to intensive care units (ICU) (*n* = 460). In adults with asthma, allergic bronchopulmonary aspergillosis (ABPA) was the second most common respiratory mycoses noticed (*n* = 30,062) along with severe asthma with fungal sensitization (*n* = 39,628). Invasive aspergillosis was estimated in 184 cases undergoing anti-cancer treatment and 834 ICU cases. Cryptococcal meningitis was diagnosed in 700 subjects with HIV/AIDS and *Pneumocystis jirovecii* pneumonitis (PCP) in 1286 subjects with underlying HIV disease. The present study indicates that at least 590,214 of the Malaysian population (1.93%) is affected by a serious fungal infection annually. This problem is serious enough to warrant the further epidemiological studies to estimate the burden of human fungal infections in Malaysia.

## 1. Introduction

Fungal infections (mycoses) lead to significant rates of morbidity and mortality worldwide, ranging from mild to invasive disease in humans, plants and animals [1]. Individuals with a dysfunctional immune system such as immunocompromised people with underlying HIV infection, very young children and the elderly are vulnerable to fungal infections [2]. Mycoses such as *Pneumocystis* pneumonia [3,4,5,6], cryptococcal meningitis [7,8,9], disseminated histoplasmosis [10,11,12] and *Talaromyces marneffei* (*formerly Penicillium marneffei*) infection [1,13] are reported commonly among patients with HIV/AIDS and may occur in those with cancer or in intensive care or with adult onset immunodeficiency [2,14]. Further, individuals with chronic respiratory disease are susceptible to airborne fungal infections including allergic bronchopulmonary aspergillosis (ABPA) [15,16], severe asthma with fungal sensitization [17,18], chronic pulmonary and invasive aspergillosis [19]. With the advent of modern diagnostic non-culture-based technology, the number of individuals diagnosed with systemic fungal infections (SFIs) will rise as more patients are diagnosed, but in addition, modern immunosuppression also brings its risks [6,20,21] and cases may rise steadily over the coming years. Estimates from Brown et al., [21] reveal that the global prevalence of SFIs has risen to 4 for every 1000 individuals of the global population. Estimates also suggest that >90% of fungal infections resulting in death are attributed to by *Candida* spp., *Cryptococcus* spp., *Aspergillus* spp. and *Pneumocystis jirovecii*—together causing over two million life-threatening infections globally, each year [19,21].

In most developing countries, national community-based surveillance programs are usually considered as the gold-standard for estimating disease prevalence and incidence. However, it is difficult for all countries to implement this uniformly as it is complex and can be very expensive. Together, these factors have contributed to the paucity of systemic data on the incidence and prevalence of human fungal infections in Malaysia. Many studies have employed sentinel surveillance to provide data at a much lower cost [22], although this approach has its own limitations, and therefore must be adjusted across urbanized and densely populated communities. Especially in Asia, where overlapping healthcare providers represent the norm [23], high quality epidemiological studies require considerable resources.

Several researchers have recently undertaken an actuarial approach to estimate the burden of fungal infections at the national level [1,8,16]. Subsequently, because of non-standardized data available on global fungal infections and the prevailing limited quantity of systematic data on the incidence and prevalence of fungal infections in Malaysia, we have aimed in this paper to try to estimate the burden of serious human fungal infections in Malaysia. The current estimation is expected to provide a better perspective of our understanding of the global distribution of serious human fungal infections, serve as a reference for future investigations, and will contribute to the improvement in the public health aspects of fungal infections in the region.

## 2. Material and Methods

The burden of serious fungal infections in Malaysia was estimated among both a general healthy population and individuals at risk including people living with HIV/AIDS, chronic obstructive pulmonary disease (COPD), asthma, cancer, tuberculosis (TB) as well as those in intensive care for treatment of chronic conditions. Since there is no specific data on serious fungal infections, the estimation of the burden of fungal infections was derived from worldwide data and based on the methodology of the Leading International Fungal Education (LIFE) program. Data on the Malaysian population were extracted from World Heath Statistic 2011 [24] based on age and gender. In addition, the epidemiology data were also extracted from various journals and articles obtained via PubMed and local journal database.

HIV population data including the number of HIV cases, proportion of HIV-diagnosed patients not receiving antiretroviral therapy (ART) and AIDS-related deaths were derived from WHO Progress Report 2011: Global HIV/AIDS Response [25] and the Jointed United Nations Programme on HIV/AIDS (UNAIDS) 2012 [26] as well as local reports for HIV. The data regarding the proportion of AIDS patients presenting with different fungal infections were derived from several epidemiological reports where cryptococcal meningitis and *T. marneffei* infection data were derived from General South East Asian rate [27,28] and Vietnam data [1,29], respectively. Pneumocystosis and histoplasmosis data were from Ratabasuwam (2005) [30] and Lian et al., (2007) [31], respectively. The incidence of oesophageal candidiasis has been estimated to occur in ~20% of HIV-infected patients without ART, and 5% of those receiving ART and so the incidence in Malaysia was estimated. The total annual incidence of pulmonary TB (PTB) was obtained from the WHO Global Tuberculosis Report [32]. Post-TB chronic pulmonary aspergillosis (CPA) was estimated based on the assumption that 22% of the patients with lung cavities and 2% of patients without cavities generally develop CPA [19,33]. Data on COPD and asthma were derived from Lim et al., (2015) [31] and To et al., (2012) [34], respectively. The prevalence of COPD in all GOLD stages was obtained [34]. The prevalence of ABPA was estimated using a rate of 2.5% of adult asthmatics [15]. Further, the prevalence of severe asthma with fungal sensitization (SAFS) was estimated to be 33% among ~10% of the total adults with the most severe and poorly controlled asthma [17].

The estimation of candidemia was performed using a general rate of 5 per 100,000 population [33,35]. We assumed that about a third of such cases occurred in intensive care, and *Candida* peritonitis (intra-abdominal candidiasis) which usually complicates complex surgery was also seen only in intensive care at a rate 50% of the candidaemia rate [36]. The number of cases of recurrent vaginal candidiasis was estimated based on the expected prevalence of 6% in women between 15–50 years old, derived from Sobel et al., (2007) [37]. The incidence of fungal keratitis was estimated based on the assumption of 10 cases per year in each of 40 hospitals. Data related to hematological malignancy and transplantation were collected depending on data availability.

## 3. Results

Malaysia has an estimated population of ~30 million people; ~29% are children and ~11.3 million are adult women, with 8% of women over 60 years of age [24]. The estimation of serious fungal infections based on the population is shown in Table 1, with the number of infections per underlying disorder and the rate per 100,000 population.

### 3.1. HIV/AIDS and AIDS-Defining Fungal Infections

According to the UNAIDS 2012 report, people living with HIV were estimated to be ~82,000, of which ~35,000 with CD4 <350 are not on any active ART regimen [25]. Opportunistic fungal infections are relatively common in HIV-infected people. Oesophageal candidiasis is a common serious fungal infection [14,33,38], and 5,850 cases of oesophageal candidiasis are estimated annually, (19 per 100,000). Cryptococcal meningitis is a common fungal infection in AIDS and among severely immunocompromised patients, usually caused by *Cryptococcus neoformans* [7,8]. A total of 700 cases of cryptococcal meningitis in AIDS were estimated in Malaysia based on an annual rate of 8% in late stage HIV infection, and an additional 155 cases, some in non-immunocompromised patients. Cryptococcal meningitis has the highest incidence and mortality rates among subjects with advanced HIV disease stages and its global burden has recently been re-estimated in AIDS—508 cases and 456 deaths in Malaysia, similar to our estimate [8]. Similarly, *Pneumocystis* pneumonia (PCP) is also an AIDS-defining illness with an estimated 1286 cases, based on a 15% rate in newly presenting patient with AIDS. PCP is the second most common fungal infections diagnosed among HIV-infected patients in Malaysia [14]. Disseminated histoplasmosis [10,14] and *T. marneffei* infection [13,14] were also reported in patients with HIV/AIDS. Here, we estimate 175 cases of disseminated histoplasmosis and 350 cases with *T. marneffei* infection in those with advanced HIV infection.

### 3.2. Pulmonary Mycoses

The annual incidence of pulmonary TB was an estimated 18,923 cases reported in 2013 [32]. Among pulmonary TB survivors, CPA has an estimated annual incidence of 1211 and prevalence of 3817. Post-TB cavitation acts as a predisposing factor for the colonisation of *Aspergillus* spp., largely owing to the development of empty residual lung cavities following treatment for TB [19]. As CPA is also found in patients with many other pulmonary disorders, and occasionally as a primary illness [39], we have assumed a total prevalence of 7635 patients. 

ABPA [15,40] and SAFS [17,41,42] are the most common fungal infections among asthmatics with a notably high prevalence rate. According to a past study, the rates of asthma in adults are 5.51 per 100,000 population, and there are 1.2 million adults with asthma in Malaysia [43]. Among the estimated population of adult asthmatics, ABPA has been estimated in 30,062 (98/100,000) and SAFS in 39,682 cases (130/100,000). ABPA is rare in children and cystic fibrosis is rare in Malaysia. The prevalence and clinical impact of SAFS in children are yet to be described.

### 3.3. Invasive Fungal Infections

Invasive aspergillosis (IA) [44,45] and candidemia [35,46,47,48] are the major invasive fungal infections in modern medicine. The latter typically being a bloodstream infection, with far reaching implications for immunosuppressed individuals. Individuals with acute leukemia or transplantation are at high risk for the development of invasive fungal infections [49,50]. Exacerbations of COPD and hospitalization, particularly to intensive care, are participating factors, as is chemotherapy for cancers and immunotherapy for transplantation. 

COPD classification was done according to the Global Initiative for Chronic Obstructive Lung Disease (GOLD) criteria. GOLD stage I, II, III, IV refer to COPD severity of mild, moderate, severe and very severe respectively. The prevalence of COPD across all GOLD stages in Malaysia was 5.1% in those 40 years and older, of whom 12.5% were of the severe symptomatic phenotype and 19% had been hospitalized [34]. A total of 1,018 cases of IA were estimated among immunocompromised patients, notably in ICU mostly with COPD as well as acute leukemia.

Meanwhile, 1073 cases of candidaemia were estimated among patients with haematological malignancies, on the medical wards or having undergone transplantation and another 460 cases were estimated to occur among subjects in ICU with an annual rate of 5 per 100,000 population [35]. This is likely to be a conservative rate, in the absence of population-based epidemiological data, and based on experience in other South East Asian countries [51]. We also estimated 230 patients with intra-abdominal candidiasis, and have not attempted to estimate *Candida* peritonitis occurring in those on chronic ambulatory peritoneal dialysis.

### 3.4. Fungal Keratitis

Fungal keratitis is a challenging ophthalmological problem leading to corneal blindness [52,53,54,55]. It commonly occurs in developing countries, including Malaysia. Ocular trauma and use of contact lenses appear to be the common etiologies leading to fungal keratitis. *Fusarium* spp. are the most common aetiological agents for fungal keratitis followed by *Aspergillus* spp. and *Candida* spp. [53]. In the current study, we estimated a total of 400 cases of fungal keratitis, a prevalence of 1.3 per 100,000 population in Malaysia. This can be further supported by the 5-year retrospective review, which showed 46% of cases of fungal keratitis due to *Fusarium* spp., and higher incidence among males [55].

### 3.5. Recurrent Vaginal Candidiasis

Vaginal candidiasis is a common gynecological problem occurring among women globally, most commonly caused by *Candida albicans,* but occasionally by other *Candida* species [37,56]. It is reported that ~70–75% of all women will experience at least one episode of vaginal candidiasis in their lifetime, especially during pregnancy [57]. Up to 40–55% of infected women will suffer from recurrent vaginal candidiasis [56]. Recurrent vaginal candidiasis is defined as at least four episodes of infection per year [37]. According to a global epidemiological review, ~5–9% women were estimated to be infected with recurrent vaginal candidiasis annually [37,57,58]. Our estimate shows that it is common in Malaysia where a total of 501,138 otherwise healthy women were estimated to be infected with recurrent vaginal candidiasis at a rate of 4800 per 100,000 females per year.

## 4. Discussion

Here, we report the estimated incidence and prevalence of certain serious fungal infections in Malaysia is approximately 2% of the population, equating to 590,214 of the total burden. Recurrent vaginal candidiasis has the highest burden of all the selected fungal infections. The second most common mycoses in Malaysia are asthma-associated fungal infections, ABPA and SAFS, although infrequently diagnosed or treated with antifungals. However, the actual cases of serious fungal infection may be higher than the current estimation as we have very little data on allergic fungal sinusitis, tinea capitis, histoplasmosis in non-AIDS patients, mucormycosis, sporotrichosis, mycetoma, chromoblastomycosis and phaeohyphomycosis. Nonetheless, this problem is serious enough to warrant more comprehensive epidemiological studies of fungal diseases in Malaysia. Further some estimates are likely significant underestimates, including *candida* bloodstream infection [51].

Serious fungal infections contribute to chronic illness and mortality across the world. In order to reduce the burden of fungal infection, a national reporting system on fungal infections and a fungal surveillance system should be implemented and improved to ascertain the impact of serious fungal infections, and promote public awareness of the seriousness of these infections. In addition, advanced laboratory diagnostic techniques and better surveillance within the healthcare system is likely to reduce the unnecessary burden of serious fungal infections in Malaysia.

Malaysia, complemented by the implementation of advanced laboratory diagnostic techniques, proper national reporting of fungal infections, and greater integration of fungal diseases management in Malaysia’s health care system, can achieve better outcomes in reducing the impact and burden of fungal diseases [59].

## Figures and Tables

**Table 1:** Estimated annual cases and total burden of serious fungal infections in Malaysia.

Fungal Infection	Number of Infections per Underlying Disorder per Year					Total Burden	Rate/100,000
	None	HIV/AIDS	Respiratory	Cancer/Tx	ICU		
Oesophageal candidiasis	-	5850	-	-	-	5850	19
Candidaemia	-	-	-	1073	460	1533	5
*Candida* peritonitis					230	230	0.8
Recurrent vaginal candidiasis (>4×/year)	501,138	-	-	-	-	501,138	4800 *
ABPA	-	-	30,062	-	-	30,062	98
SAFS	-	-	39,682			39,682	130
Chronic pulmonary aspergillosis	-	-	7635	-	-	7635	24.9
Invasive aspergillosis	-	-		184	834	1018	3.3
Cryptococcal meningitis	47	700	-	108	-	855	2.8
Pneumocystis pneumonia	-	1286	-	-	-	1286	4.2
Histoplasmosis		175				175	0.6
*T. marneffei* infection		350				350	1.1
Fungal keratitis	400					400	1.3
Total burden estimated	501,585	8361	77,379	1365	1524	590,214	

Tx, transplant recipients; ICU, intensive care unit; ABPA, allergic bronchopulmonary aspergillosis; SAFS, severe asthma with fungal sensitization; HIV/AIDS, Human immunodeficiency virus infected/Acquired immunodeficiency syndrome; * Note rate of recurrent vaginal candidiasis is per 100,000 females.
